# Elderly Onset Spondyloarthropathy and VEXAS Syndrome: A Case Report

**DOI:** 10.31138/mjr.271223.eos

**Published:** 2024-09-30

**Authors:** Harsh Jain, Debaditya Roy, Sunil Mavidi, Subhankar Haldar, Sumantro Mondal, Paramita Bhattacharya, Alakendu Ghosh

**Affiliations:** 1 Department of Clinical Immunology and Rheumatology, Institute of Postgraduate Medical Education and Research, Kolkata, West Bengal, India; 2 Genetics Service Unit, National Institute of Biomedical Genomics, Kalyani, India

**Keywords:** spondyloarthropathy, VEXAS Syndrome, macrocytic anaemia

## Abstract

We report the case of a 67-year-old male with a two-year history of inflammatory polyarthritis, fatigue, and low back pain. He also had a history of biopsy proven neutrophilic dermatosis in the past. On admission and examination, he had pallor. Laboratory evaluation showed macrocytic anaemia, elevated erythrocyte sedimentation rate (ESR), and C-reactive protein (CRP). MRI of Sacroiliac joints showed presence of bilateral sacroiliitis. Bone marrow examination showed the presence of cytoplasmic vacuolisation in myeloid and erythroid precursor cells. Genetic analysis confirmed a diagnosis of VEXAS syndrome. He improved with prednisolone and Sulfasalazine with no further relapse on follow up. This case report highlights the importance of considering VEXAS syndrome in older adults with presentation of spondyloarthritis and macrocytic anaemia. Early diagnosis and treatment with corticosteroids and steroid-sparing agents can lead to significant improvement in symptoms and are important for a good outcome.

## INTRODUCTION

VEXAS Syndrome is a recently discovered monogenic, adult-onset, autoinflammatory syndrome associated with loss-of-function mutations in codon 41 of UBA1, encoding E1- ubiquitin ligase. Most of the reported VEXAS mutations occur at codon 41 of UBA1.^1^ It may be a prototype for a new class of “haemato-inflammatory diseases”, including macro-cytic anaemia, thrombocytopenia, thromboembolic disease, and bone marrow failure, which can evolve into haematologic malignancy.^1^

Although musculoskeletal symptoms have been reported previously in literature in patients with VEXAS Syndrome,^2^ occurrence of spondyloarthritis remains extremely rare with very few patients documented globally with this clinical phenotype associated with VEXAS Syndrome. Here, we report a such rare case, along with a literature review.

## CASE-BASED DESCRIPTION

A 67-year-old male presented to our clinic with inflammatory polyarthritis, fatigue, and low back pain for 2 years. He gave a past history of multiple erythematous papules and plaques over the bilateral upper limb, trunk, and back, lasting for one month for which he took a dermatology opinion. Skin biopsy of the rash revealed neutrophilic dermatosis- suggestive of Sweet syndrome. The rash responded to a short course of oral prednisolone (Dose: 0.5 mg/kg body weight) tapered over a month with no relapse of lesions. There were no other systemic features except back pain and inflammatory polyarthritis.

**Figure 1.**
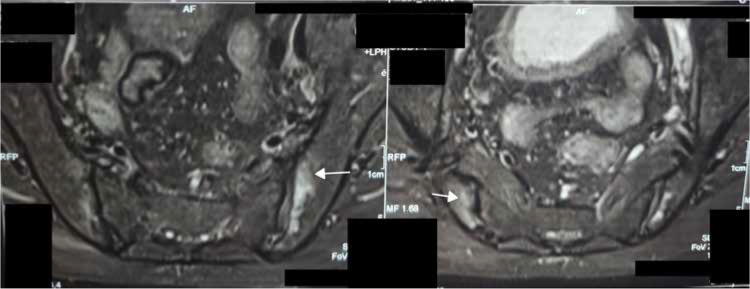
MRI of the sacroiliac joints in STIR sequence showing bilateral sacroiliitis characterised by increased signal intensity depicting bone marrow oedema in bilateral sacroiliac joints.

On current evaluation, HLA-B27 was positive, and magnetic resonance imaging (MRI) of the sacroiliac (SI) joints showed bilateral active sacroiliitis **(Figure 1)**. Musculo-skeletal ultrasound showed bilateral wrist joint synovitis and bilateral retrocalcaneal bursitis. Patient was fulfilling the Assessment of Spondyloarthritis International Society (ASAS)^3^ Criteria for the diagnosis of Axial Spondyloarthropathy and started on Sulfasalazine 1 gram per day along with non-steroidal anti-inflammatory drugs (NSAIDs). Over the next 3 months due to suboptimal response, oral Methotrexate 15 mg once a week was added with folate. In view of persistent symptoms, he was admitted for evaluation. On admission, he had pallor and tender bilateral elbow and shoulder joints, and entheseal tenderness. Systemic examination was unremarkable. Evaluation showed anaemia with haemoglobin of 8.3 gm/dl, Mean Corpuscular Volume (MCV) 106 fl, serum iron – 19 microgram/dl; total iron binding capacity of 273 microgram/dl, ferritin- 389 microgram/dl.

**Figure 2.**
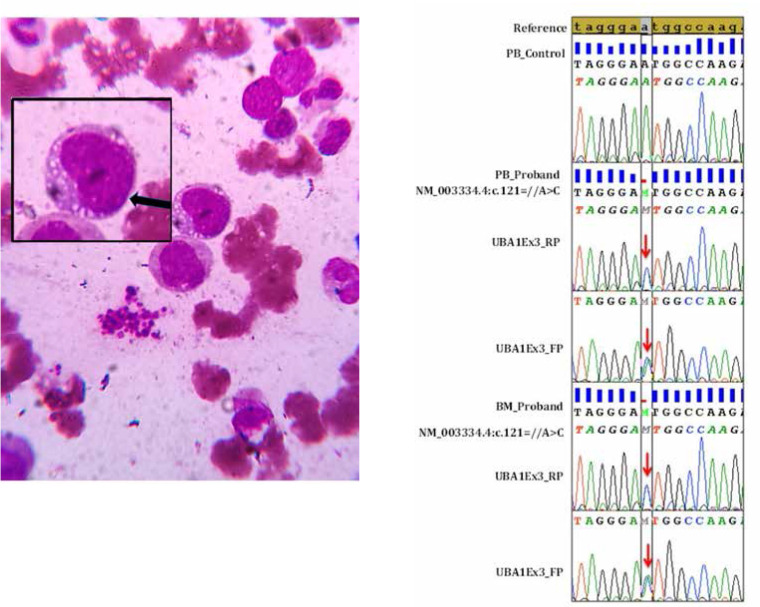
**(A)** Bone marrow biopsy showing presence of cytoplasmic vacuolisation in myeloid and erythroid series. **(B)** Sequence chromatograph showing presence of c.121A>C variant (indicated by red arrow) along with wild type allele (A) in DNA isolated from both peripheral blood (PB) and bone marrow (BM) samples of Proband compared to presence of only wild type allele (A) in peripheral blood (PB) sample of control.

Vitamin B12 and folic acid levels were normal. Inflammatory markers were raised with Erythrocyte sedimentation rate (ESR) −136 mm/hour (Normal-<20 mm/hour) and C-Reactive Protein (CRP)-38 mg/L (Normal-<6 mg/L). Upper gastrointestinal endoscopy and colonoscopy were normal. Methotrexate and Sulfasalazine were discontinued due to the possibility of drug-related macrocytosis.^4,5^ Bone marrow examination showed the presence of cytoplasmic vacuolisation in myeloid and erythroid precursor cells **(Figure 2A)**. In view of the above clinical picture and bone marrow findings, Vacuoles, E1 enzyme, X-linked, Autoinflammatory, Somatic (VEXAS) Syndrome was suspected. Genetic analysis by bidirectional Sanger sequencing and capillary electrophoresis showed pathogenic missense SNV c.121A>C (p.Met 41Leu) in exon 3 of X-linked UBA1 (NM_003334.4) gene along with the wild-type allele in peripheral blood and bone marrow samples; confirmed diagnosis of VEXAS Syndrome **(Figure 2B)**. He was initiated on oral prednisolone 10 mg with gradual tapering along with Sulfasalazine 2 gram daily.

He had good response to treatment with improvement of articular symptoms and systemic features with no further relapse (follow-up of 12 weeks) and currently on prednisolone 2.5 mg and Sulfasalazine 1 gm/day.

## DISCUSSION

In our case, a diagnosis of VEXAS was confirmed through genetic testing with patient initially having cutaneous and musculoskeletal symptoms, followed by haematological involvement in due clinical course.

While exploring literature on VEXAS, clinical reviews from various cohorts,^2^ showed that cutaneous involvement were most common (∼83.6%), followed by constitutional symptoms (∼65%), haematological involvement (∼50–62.5%), lung involvement (∼50%), musculoskeletal symptoms (∼27–46%), ocular symptoms (∼40.5%), cardiovascular system (∼11%; having arterial involvement in ∼10.3% and venous thrombosis in ∼10- 56%), lymphadenopathy (∼34.5%), splenomegaly (∼13.8%), gastrointestinal involvement (∼13.8%), and nervous system involvement (∼2.6–5.2%).^2,6^

Arthralgia and myalgia were more common symptoms than mono-, oligo-, or poly-arthritis. Interestingly, up to 50% of patients developed inflammation of the cartilage in the ears, nose, and other parts of the body and were diagnosed as VEXAS-associated relapsing polychondritis (VEXAS-RP).^6^

In a study by Obiorah et al.,^7^ all 16 patients enrolled had macrocytic anaemia. Vacuoles were found in the early precursors of red blood cells (blasts, promyelocytes, and pronormoblasts). Overall, 15% of myeloid and erythroid cells had vacuoles, with an average of 5–7 vacuoles per cell. Lymphocytes were generally devoid of vacuoles. Dermatological manifestations that have been reported are neutrophilic dermatoses, vasculitis, and periorbital angioedema. Dermal infiltrates are proposed to be derived from the pathological myeloid clone.

Spondyloarthritis remains a very rare phenotype in VEXAS syndrome with only a few cases reported in the literature.^8^ Occurrence of B27 alleles has been reported and may represent an epistatic enhancement to specific clinical manifestations of VEXAS syndrome, with features typical of Spondyloarthritis.^9^ One of the probable evolution of the Spondyloarthropathy phenotype in VEXAS syndrome is due to somatic mutations affecting methionine-41(p.Met41) in UBA1 resulting in decreased ubiquitylation and activated innate immune pathways.^8,9^ In animal models, ubiquitin-editing enzyme A20 prevented spondyloarthritis by restricting TH17 cell expansion.^8,9^ Treatment involves steroids and steroid-sparing agents like anti-IL1 inhibitors, anti-IL6, and Janus Kinase (JAK) inhibitors.

In the multicentre French Cohort,^2^ VEXAS phenotype was also seen associated to type of UBA1 mutation, i.e. a phenotype-genotype correlation. It was observed that patients with the UBA1 p.Met41Leu mutation were less likely to experience fever (19%) or lung involvement (9.5%) and were less likely to have unprovoked thrombosis than those with the UBA1 p.Met41Val mutation. Patients with the UBA1 p.Met41Val mutation were more likely to experience chondritis (14.3%), higher CRP levels, and more frequent myelodysplastic syndrome (MDS) (68.6%) and had a decreased survival rate.^2^ Interestingly, the UBA1 p.Met41Leu mutation was more common in patients with mild-to-moderate disease (29.6%) as were the non-Met-41 variants.^10^ This can also be observed in our patient, who has the p.Met41Leu mutation, which may predict that his disease will have a indolent course compared to the other more aggressive forms of this condition. To the best of our knowledge, this is the first reported case of VEXAS syndrome from Eastern India and the first reported case of VEXAS associated with elderly onset spondyloarthropathy from India. Deciphering this patient’s illness and connecting the symptoms and signs was a diagnostic puzzle. Eventually they pointed to both spondyloarthritis and a newly discovered syndrome, VEXAS. Age added another layer of complexity. Detailed workup finally unlocked the answer. Further link between these two conditions needs to be proven in subsequent studies.

In conclusion, timely recognition of clinical patterns along with genetic testing in an older male with Spondyloarthritis, macrocytic anaemia and neutrophilic dermatosis prompted us to suspect VEXAS with eventual diagnosis and treatment. In the future, phenotype-genotype correlation may shed more light on VEXAS, predict and prognosticate its clinical course, and how to best treat and manage this challenging and rare condition.

## KEY MESSAGE

An association with VEXAS syndrome should be considered while evaluating an elderly male with spondyloarthropathy and macrocytic anaemia.
